# Analysis of combined resistance to oxazolidinones and phenicols among bacteria from dogs fed with raw meat/vegetables and the respective food items

**DOI:** 10.1038/s41598-019-51918-y

**Published:** 2019-10-29

**Authors:** Yifan Wu, Run Fan, Yinchao Wang, Lei Lei, Andrea T. Feßler, Zheng Wang, Congming Wu, Stefan Schwarz, Yang Wang

**Affiliations:** 1 Beijing Advanced Innovation Center for Food Nutrition and Human Health, College of Veterinary Medicine, China Agricultural University, Beijing, 100193 P.R. China; 2 Institute of Microbiology and Epizootics, Centre for Infection Medicine, Department of Veterinary Medicine, Freie Universität Berlin, 14163 Berlin, Germany

**Keywords:** Antimicrobial resistance, Bacterial genomics

## Abstract

The gene *optrA* is the first gene that confers resistance to the oxazolidinone tedizolid, a last resort antimicrobial agent in human medicine. In this study we investigated the presence of *optrA* and the multi-resistance genes *poxtA* and *cfr* in enterococci and staphylococci from (i) pet animals known to be fed raw meat and vegetables and (ii) the respective food items. We examined 341 bacterial isolates from cats and dogs, 195 bacterial isolates from supermarket food items and only one *E. faecium* collected from industrial food in Beijing during 2016. Thirty-five (6.5%) of the 537 isolates, including 31/376 (8.2%) enterococci and 4/161 (2.5%) staphylococci, were positive for *optrA*, while all isolates were negative for *poxtA* and *cfr*. S1-nuclease pulsed-field gel electrophoresis (PFGE) and Southern blotting confirmed that *optrA* was located in the chromosomal DNA of 19 isolates and on a plasmid in the remaining 16 isolates. Whole genome sequencing revealed several different genetic environments of *optrA* in plasmid- or chromosome-borne *optrA* genes. PFGE, multilocus sequence typing (MLST) and/or SNP analysis demonstrated that the *optrA*-carrying *Staphylococcus* and *Enterococcus* isolates were genetically heterogeneous. However, in single cases, groups of related isolates were identified which might suggest a transfer of closely related *optrA-*positive *E. faecalis* isolates between food items and dogs.

## Introduction

The abbreviation BARF stands for ‘Biologically Appropriate Raw Food’ and describes a way of nutrition of dogs and cats by which exclusively natural food (including raw meat, entrails and bones) is fed to the respective animals^1^. This diet is often supplemented by the addition of raw vegetables, fruits, nuts and cold-pressed oils. Cooked food and conventionally available dog and cat food are not fed at all. By applying the BARF method, animal owners try to imitate the natural nutrition of wolves and feral dogs and cats. Natural food – in contrast to industrial pet food – bears a higher risk of contamination with antimicrobial resistant bacteria. In this regard, eating raw meat has been identified as a risk factor for the carriage of ampicillin-resistant *Enterococcus faecium* in dogs in the Netherlands^2^.

Antimicrobial resistance is currently one of the greatest threats to global health as well as food safety^3^. It refers to bacteria from humans, animals and environmental sources, among which resistances to virtually all classes of antimicrobial agents may occur. Although last resort antimicrobial agents used in human medicine, such as the oxazolidinones, are not approved for use in food-producing animals and are rarely used in companion animals, resistance to these drugs has been encountered in bacteria of animal origin. Linezolid, the first oxazolidinone introduced into clinical use in 2000, is still considered a highly efficient antimicrobial agent against clinically important Gram-positive pathogens, including methicillin-resistant staphylococci and vancomycin-resistant enterococci^3,4^. However, linezolid-resistant *Enterococcus* and *Staphylococcus* isolates have been reported since the year 2000^4^. Linezolid resistance in these two genera has often been associated with mutations in the central loop of domain V of the 23S rRNA, as well as in ribosomal proteins L3, L4 and L22^5–7^. The first reported transferable oxazolidinone resistance gene, *cfr*, mediates resistance not only to linezolid, but also to phenicols, lincosamides, pleuromutilins, and streptogramin A type antibiotics by encoding a methyltransferase that modifies the 23S rRNA at position A2503^8^. Since *cfr* was first identified in bovine *Staphylococcus sciuri*, it has also been reported in various *Staphylococcus* species from humans, pets, and farm animals^4,9–11^. Recently, a *cfr* homolog, *cfr*(B), has been discovered in *Clostridium difficile* and *Enterococcus faecium* strains in Europe^12,13^, while *cfr*(C) was identified in *Campylobacter coli* and *Clostridium difficile*^14–16^.

The second generation oxazolidinone tedizolid demonstrates improved activity against multidrug-resistant Gram-positive bacteria, including *cfr*-harboring isolates^17,18^. In 2015, we characterized a novel transferable resistance gene, *optrA*, in *Enterococcus* spp. of both human and animal origin^19,20^. The gene *optrA* codes for an ABC-F protein that confers cross-resistance to phenicols and oxazolidinones, including tedizolid^21^. A recent study showed that ABC-F proteins confer antibiotic resistance by interacting with the ribosome and displacing the drug from its binding site, thereby protecting the ribosome^21^. Recently, a new gene, named *poxtA*, which confers resistence to oxazolidinones, phenicols and tetracyclines was detected in MRSA^22^ and enterococci^23^.

In the present study, we collected anal and nasal samples from dogs and cats with a history of being fed raw meat and vegetables purchased from supermarkets, and also collected raw meat and vegetable samples from supermarkets in Beijing. We then screened florfenicol-resistant enterococci and staphylococci recovered from the respective samples for the presence of *optrA*, *poxtA* and *cfr*, and further characterized the genotypes of these isolates in order to see how related they are.

## Results and Discussion

### Analysis of resistance mechanisms to oxazolidinones and phenicols

A total of 450 anal and nasal samples were collected from dogs and cats and 187 enterococcal isolates and 154 staphylococcal isolates were recovered from these samples (Supplementary Fig. 1). An additional 224 samples were collected from vegetables and raw meat products from 28 supermarkets in Beijing, resulting in the isolation of 188 enterococcal isolates and seven *Staphylococcus aureus* isolates (Supplementary Fig. 1). From the 35 samples collected from industrial pet food, only one *E. faecium* isolate was recovered. Resistance to florfenicol was detected in 12.8% of the *Enterococcus* isolates (24/187; 23 *Enterococcus faecalis* and a single *Enterococcus faecium*) and 2.6% of the *Staphylococcus* isolates (4/154; four *S. sciuri*) from companion animals. Florfenicol resistance was also identified in 6.4% of the *Enterococcus* isolates (12/188; nine *E. faecalis* and three *Enterococcus casseliflavus*), but in none of the seven *S. aureus* isolates recovered from supermarket vegetables and raw meat samples. In addition, the single *E. faecium* recovered from industrial pet food was susceptible to florfenicol. PCR-based amplification of *optrA*, *cfr*, and *poxtA* indicated that 6.5% (35/537) of all enterococci and staphylococci and 87.5% (35/40) of the florfenicol-resistant isolates harbored *optrA*, including 28 *E. faecalis*, three *E. casseliflavus*, and four *S. sciuri* isolates. All florfenicol-resistant isolates were also positive for the phenicol exporter gene *fexA*, whereas none of the florfenicol-resistant isolates harbored *cfr*, *cfr*(B), *cfr*(C), *poxtA*, or *fexB* (Table 1). None of the isolates in this study revealed the presence of oxazolidinone resistance-mediating mutations in domain V of the 23S rRNA gene or in the genes coding for ribosomal proteins L3, L4, and L22.

**Table 1:** Characterization of 35 *optrA*-carrying isolates from companion animals and food products.

Isolates	Origin	Samples	Location of *optrA* (kb)^a^	Other CHL/FFC resistance genes	ST type	PFGE pattern	Oxazolidinone MICs (µg/ml)		Other resistance phenotype^b^
							LZD	TZD	
***E. faecalis***									
72AC	Dog	anal swab	P (~90)	*fexA*	170	K1	8	2	FFC-CHL-MIN-ERY
37AC	Dog	anal swab	P (~90)	*fexA*	170	K2	8	1	FFC-CHL-MIN-ERY
114AC	Dog	anal swab	P (~100)	*fexA*	773	A1	8	2	FFC-CHL-MIN-ERY
100AC	Dog	anal swab	P (~90)	*fexA*	22	A2	8	2	FFC-CHL-MIN-ERY
190AC	Dog	anal swab	P (~60)	*fexA*	16	G1	8	1	FFC-CHL-MIN-ERY-GEN-RIF
192NC	Dog	nasal swab	P (~60)	*fexA*	16	G2	8	1	FFC-CHL-MIN-ERY-GEN-RIF
182NS	Dog	nasal swab	P (~60)	*fexA*	16	H	4	1	FFC-CHL-MIN-ERY-GEN-RIF
121NS	Dog	nasal swab	C	*fexA*	256	I1	4	0.5	FFC-CHL-MIN-ERY-CIP-GEN
233NC	Dog	nasal swab	C	*fexA*	256	I2	8	1	FFC-CHL-MIN-ERY-CIP-GEN
3-8	Beef	washing fluid	P (~60)	*fexA*	16	I3	4	1	FFC-CHL-ERY-CIP
61NC	Dog	nasal swab	P (~90)	*fexA*	480	P1	8	1	FFC-CHL-MIN-ERY-CIP-GEN-RIF
68AC	Dog	anal swab	C	*fexA*	480	P2	8	0.5	FFC-CHL-MIN-ERY-CIP-GEN
109AC	Dog	anal swab	C	*fexA*	480	P3	2	0.25	FFC-CHL-MIN-ERY-CIP-GEN
52AC	Dog	anal swab	C	*fexA*	476	J1	2	0.25	FFC-CHL-MIN-ERY-CIP-GEN
203NC	Dog	nasal swab	C	*fexA*	476	J2	2	0.5	FFC-CHL-MIN-ERY-CIP-GEN
22-4	Chicken wing	washing fluid	C	*fexA*	476	J3	4	0.25	FFC-CHL-MIN-ERY-CIP-GEN
11-7	Egg	washing fluid	C	*fexA*	474	M1	4	1	FFC-CHL-MIN-ERY-CIP-GEN-RIF
11-8	Beef	washing fluid	C	*fexA*	474	M2	4	1	FFC-CHL-MIN-ERY-CIP-GEN-RIF
207AE	Dog	anal swab	P (~90)	*fexA*	59	M3	4	1	FFC-CHL-MIN-ERY-GEN
8-2	Caraway seeds	washing fluid	P (~60)	*fexA*	632	O1	16	1	FFC-CHL-MIN-CIP-RIF
8-3	Pork	washing fluid	P (~60)	*fexA*	632	O2	16	1	FFC-CHL-MIN-CIP-RIF
67AC	Dog	anal swab	C	*fexA*	474	N	2	0.5	FFC-CHL-MIN-ERY-CIP-GEN
82AC	Dog	anal swab	P (~100)	*fexA*	116	E	8	2	FFC-CHL-MIN-ERY
99AE	Dog	anal swab	C	*fexA*	256	C	8	1	FFC-CHL-MIN-ERY-CIP-GEN
131AC	Dog	anal swab	P (~60)	*fexA*	86	L	8	4	FFC-CHL-MIN-ERY-CIP-GEN
75AC	Dog	anal swab	P (~90)	*fexA*	230	D	4	0.5	FFC-CHL-MIN-ERY-GEN
27-3C	Pork	washing fluid	C	*fexA*	256	F	8	1	FFC-CHL-MIN-ERY-GEN
5-6	Cucumber	washing fluid	P (~90)	*fexA*	309	B	16	1	FFC-CHL-MIN-CIP
***E. casseliflavus***									
10-1	Onion	washing fluid	C	*fexA*	—	Q	4	0.5	FFC-CHL-MIN-ERY-CIP-RIF
6-8	Beef	washing fluid	C	*fexA*	—	R	4	1	FFC-CHL-DPC-RIF
25-4C	Chicken wing	washing fluid	C	*fexA*	—	S	4	0.5	FFC-CHL-ERY-DPC
***S. sciuri***									
207NS	Dog	nasal swab	C	*fexA*	—	T	4	0.5	FFC-CHL-ERY
200NS	Dog	nasal swab	C	*fexA*	—	U1	4	0.25	FFC-CHL-TIA
210NS	Dog	nasal swab	C	*fexA*	—	U2	2	1	FFC-CHL-TIA
53NC	Dog	nasal swab	C	*fexA*	—	V	4	0.25	FFC-CHL-AMP-ERY-CLI-TIA

^a^P, plasmid; C, chromosomal DNA.

^b^CHL, chloramphenicol; FFC, florfenicol; LZD, linezolid; TZD, tedizolid; MIN, minocycline; ERY, erythromycin; GEN, gentamicin; DPC, daptomycin; CLI, clindamycin; TIA, tiamulin; AMP, ampicillin; CIP, ciprofloxacin; RIF, rifampicin.

### Antimicrobial susceptibility profiles

All *optrA*-positive enterococci from companion animals originated from dogs, were resistant to florfenicol, chloramphenicol, and erythromycin, and exhibited high MICs to virginiamycin M1. Most of these isolates also exhibited resistance to minocycline (95%), as well as high-level gentamicin resistance (75%). Furthermore, about half of these isolates were resistant to ciprofloxacin. However, all *optrA*-positive enterococci from companion animals were susceptible to vancomycin, daptomycin, and ampicillin. All *optrA*-positive enterococci isolated from food were resistant to florfenicol and chloramphenicol. Most of these isolates also showed resistance to minocycline (72.7%), ciprofloxacin (72.7%), exhibited high MICs to virginiamycin M1 (72.7%), and all were susceptible to ampicillin and intermediate to vancomycin (Table 1).

All *optrA*-positive staphylococci originated from dogs and were identified as *S. sciuri*. They were resistant to florfenicol, chloramphenicol and oxacillin. Most of these isolates also exhibited resistance to erythromycin (60%) and high MICs to tiamulin (60%), but all were susceptible to vancomycin, minocycline, daptomycin, gentamicin, and rifampicin (Supplementary Table 1). The presence of resistant bacteria did not differ significantly between the samples from pets and supermarket-derived food items, except for those showing resistance to minocycline and erythromycin (Supplementary Table 2). A previous study from Denmark identified three *optrA*-positive enterococci isolated from food products that had a similar resistance pattern as the isolates in the current study, showing resistance to ciprofloxacin, erythromycin, aminoglycosides, and tetracycline^24^.

The MICs of linezolid and tedizolid of the *optrA*-positive staphylococci and enterococci were in the range of 2–16 µg/ml and 0.25–4 µg/ml, respectively. Among the 35 isolates, 30 were classified as non-susceptible to linezolid (4–16 µg/ml), with 22 of them being also classified as non-susceptible to tedizolid (≥1 µg/ml). The percentage of non-susceptibility to linezolid among enterococcal isolates from pets in the present study (8.56%, 16/187) is relatively high compared with previous studies conducted in Canada (0.40%), Korea (0.33%), and China (2.03%)^25–27^. Linezolid use in animals is banned in China, and phenicol antibiotics are rarely used at the China Agricultural University Veterinary Teaching Hospital for the treatment of dogs.

### Location and characteristics of *optrA*

S1-PFGE and Southern blotting revealed that *optrA* was located in the chromosomal DNA of 12 *E. faecalis* isolates and on a plasmid in the remaining 16 isolates. In the three *E. casseliflavus* isolates and the four *S. sciuri* isolates, *optrA* was located in the chromosomal DNA (Supplementary Fig. 2). Table 2 shows that all isolates with linezolid MICs of 2 µg/ml have chromosomal *optrA* genes and all five isolates with tedizolid MICs of 0.25 µg/ml and seven of the eight isolates with tedizolid MICs of 0.5 µg/ml have chromosomal *optrA* genes. This observation may suggest that a chromosomal location of *optrA* may be associated with lower oxazolidinone MICs. However, the OptrA variant present in the respective isolates also needs to be taken into account.

**Table 2:** Polymorphisms in the OptrA proteins detected in the 35 *optrA*-positive *Enterococcus* and *Staphylococcus* isolates in relation to the chromosomal or plasmidic location of the *optrA* genes and the MICs of linezolid and tedizolid of the corresponding isolates.

OptrA variant	Mutations compared with OptrA_E349_ from *E. faecalis* E349^a^	Isolates	Origin	Location of *optrA*	Oxazolidinone MICs (µg/ml)	
					LZD	TZD
OptrA_E349_	No mutations	121NS	Dog	C	4	0.5
		233NC	Dog	C	8	1
		68AC	Dog	C	8	0.5
		99AE, 190AC, 192NC	Dog	C	8	1
D	Tyr176Asp	67AC	Dog	C	2	0.5
		11-7, 11-8	Food	C	4	1
		182NS	Dog	P	4	1
DD	Tyr176Asp, Gly393Asp	52AC, 203NC	Dog	C	2	0.25
		27-3C	Dog	C	8	1
		25-4C	Dog	C	4	0.5
		53NC	Dog	C	4	0.25
		22-4	Food	C	4	0.25
		10-1	Food	C	4	0.5
DP	Tyr176Asp, Thr481Pro	75AC	Dog	P	4	0.5
		3-8	Food	P	4	1
DM	Tyr176Asp, Ile622Met	109AC	Dog	C	2	0.25
		6-8	Food	C	4	1
		131AC	Dog	P	8	4
DNDM	Tyr176Asp, Asp247Asn, Gly393Asp, Ile622Met	207NS	Dog	C	4	0.5
		210NS	Dog	C	2	1
KD	Thr112Lys, Tyr176Asp	72AC, 82AC	Dog	P	8	2
		37AC	Dog	P	8	1
		5-6	Food	P	16	1
RDK	Ile104Arg, Tyr176Asp, Glu256Lys	114AC	Dog	P	8	2
		8-3	Food	P	16	1
RD	Ile104Arg, Tyr176Asp	100AC	Dog	P	8	2
		8-2	Food	P	16	1
KDP	Thr112Lys, Tyr176Asp, Thr481Pro	61NC	Dog	P	8	1
		207AE	Dog	P	4	1
YDD	Asn12Tyr, Tyr176Asp, Gly393Asp	200NS	Dog	C	4	0.25

^a^D, Asp; K, Lys; M, Met; N, Asn; P, Pro; R, Arg; Y, Tyr.

Comparison of the deduced OptrA amino acid sequences of the 35 isolates with the OptrA of *E. faecalis* E349 (designated as the wild-type) revealed the presence of the wild-type OptrA and ten OptrA variants, all of which differed in at least one amino acid position from the wild-type (Table 2), and all these variants also differed distinctly from the variant OptrA_E35048_ found in Italy^28^. OptrA variants D, DD, DP, KD, RDK and KDP have previously been detected in large screening studies on enterococci of human and animal origin^20,29,30^. Here, we report four new variants: DM, RD, YDD and DNDM. The variants DM and RD were detected in samples from dogs and food, whereas the variants YDD and DNDM were detected only in three *S. sciuri* isolates from nasal samples of dogs. Based on the associated MIC values for linezolid and tedizolid, it is possible that the different OptrA variants have an impact on the relative oxazolidinone susceptibility of the corresponding isolates (Table 2).

### Genetic environment of plasmid-borne *optrA*

**Figure 1:**
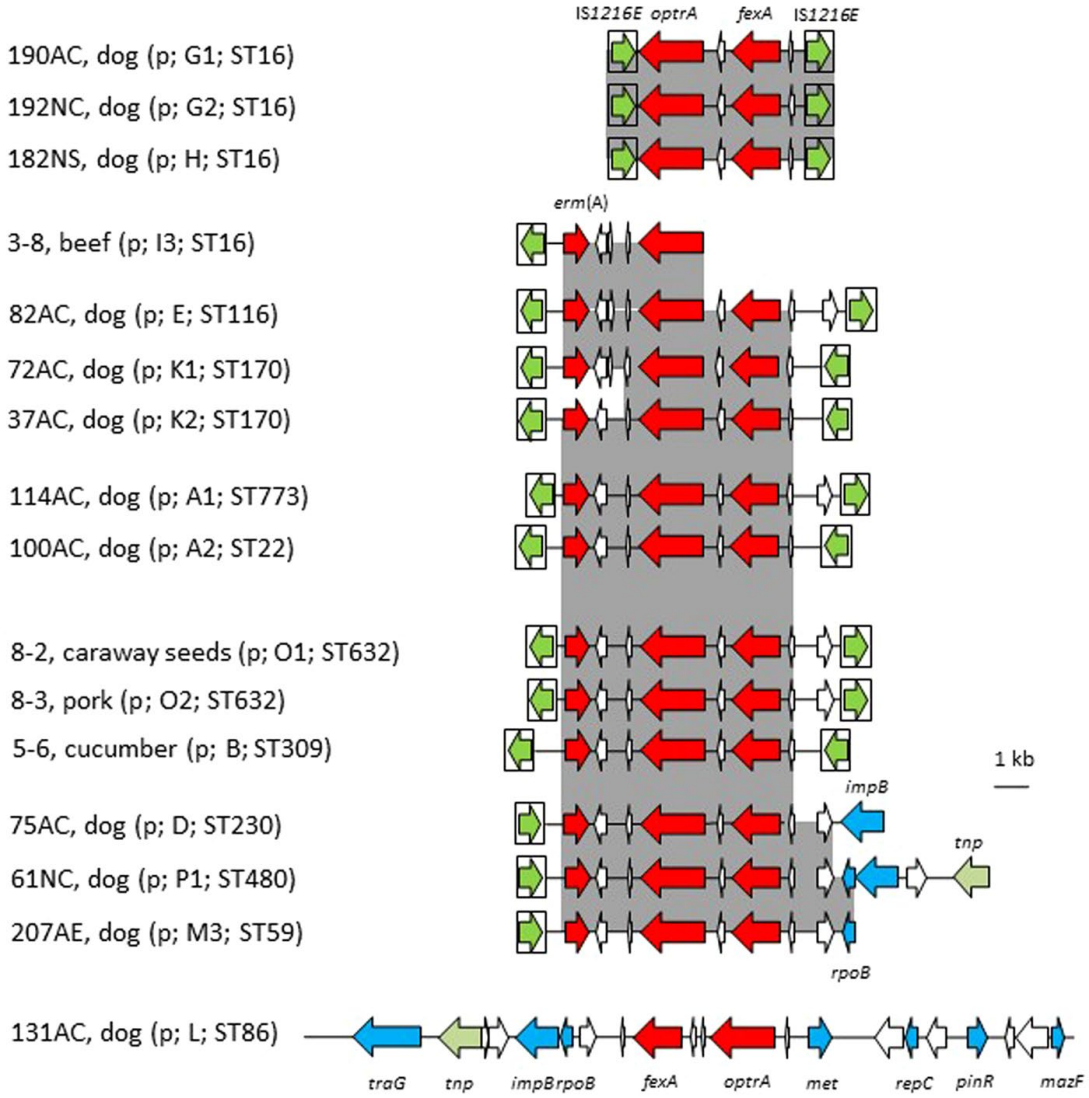
Schematic representation of the genetic environment of plasmid-borne *optrA* genes of the 16 *E. faecalis* isolates investigated in this study. Shaded areas represent regions of >99% nucleotide sequence identity. Arrows indicate the positions and orientations of the genes. White arrows represent genes coding for hypothetical proteins, red arrows indicate antimicrobial resistance genes, and blue arrows genes with other known functions. The IS*1216* elements are shown as a box with the green arrow inside indicating the transposase gene. The information in the brackets includes the location of gene *optrA* (p, plasmid), PFGE pattern, and the MLST type (both according to Table 1). ST773 (1-7-10-1-1-84-1) is a single locus variant of ST22 (1-7-10-1-1-10-1).

Sixteen *E. faecalis* isolates harbored *optrA* on plasmids ranging in size between 60–100 kb. Most of these plasmids carried the same core *erm*(A)*-optrA-fexA* resistance gene cluster (Fig. 1). Furthermore, the insertion sequence IS*1216E* was found either downstream and/or upstream of *optrA* in all plasmids except that of isolate 131AC, in similar *optrA* environments as previously reported in plasmids pSS85, pSS92, pSS79, and pFX13^29,31^. Among the plasmids with two copies of IS*1216E* bracketing the *optrA*-carrying central region, these copies were located in opposite orientations on the plasmids from isolates 8-3, 8-2, 114AC and 82AC, but in the same orientation in the other seven plasmids. When located in the same orientation, recombination between the two copies of IS*1216E* may form minicircles that contain the bracketed region plus one copy of IS*1216E*^29^, which could accelerate the transmission of *optrA*. Inverse PCR confirmed that minicircles of isolates 190AC, 192NC and 182NS were detectable, which contained the central region and one intact IS*1216E* (data not shown). This observation indicated that the IS*1216E*-flanked segment was unstable in these plasmids. Only one copy of IS*1216E*, located in the region downstream of *optrA*, was identified on the plasmids of isolates 75AC, 61NC and 207AE.

Moreover, four groups of plasmid-bearing isolates – 72AC and 37AC; 100AC and 114AC; 190AC, 192NC and 182NS as well as 8-2 and 8-3 – were identified. Within each group, the isolates shared the same or closely related MLST types, had similar PFGE patterns, shared the same resistance pattern, and carried closely related *optrA* gene regions on similar-sized plasmids (Table 1, Fig. 1). Their close genetic relationships were also confirmed by SNP analysis (Supplementary Fig. 3). All these characteristics suggested that the respective isolates are related.

### Genetic environment of chromosomal *optrA*

**Figure 2:**
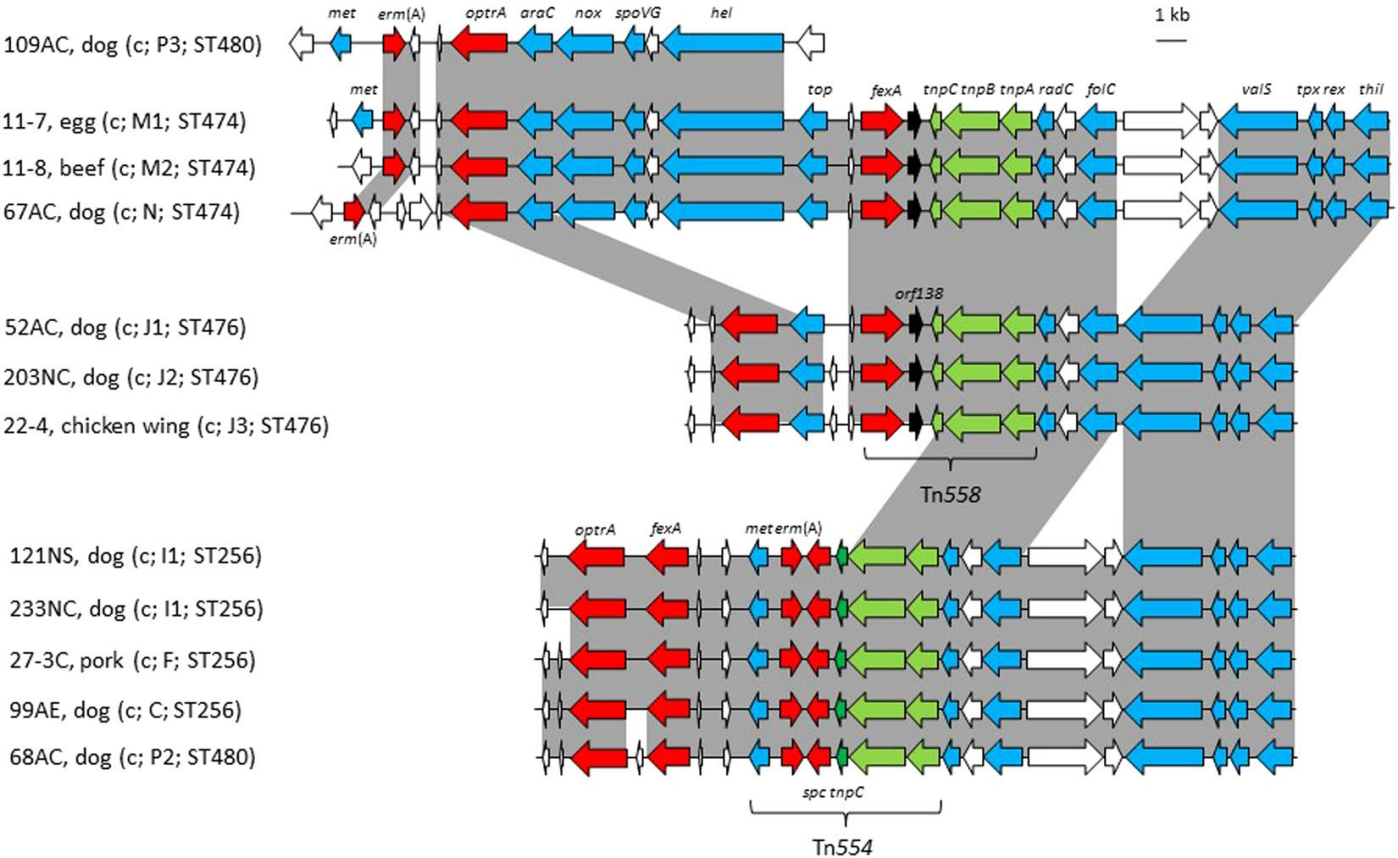
Schematic representation of the genetic environment of chromosomally located *optrA* in the twelve *E. faecalis* isolates investigated in this study. Shaded areas represent regions of >99% nucleotide sequence identity. Arrows indicate the positions and orientations of the genes. White arrows represent genes coding for hypothetical proteins, red arrows those coding for antimicrobial resistance genes, green arrows those coding for transposases, and blue arrows those coding for other known functions. The information in the brackets includes the location of gene *optrA* (c, chromosomal DNA), PFGE pattern, and the MLST type (both according to Table 1). Genes with known functions coded for a putative NADH oxidase (*nox*), the septation protein SpoVG (*spoVG*), a helicase (*hel*), a topoisomerase (*top*), a folylpolyglutamate synthase (*folC*), a valine-tRNA ligase (*valS*), a thiol peroxidase (*tpx*), a redox-sensing transcriptional repressor (*rex*), and a thiamine-phosphate kinase (*thil*). The *radC* gene codes for a DNA repair protein and represents the preferential chromosomal integration site for Tn*558* and Tn*554*.

The regions flanking *optrA* in the chromosomal DNA (Fig. 2) differed distinctly from those on plasmids. The putative transcriptional regulator *araC* was located immediately upstream of *optrA* in seven *E. faecalis* and three *E. casseliflavus* isolates. The transposon Tn*558*, which includes the genes *fexA*, *orf138*, and three transposase genes, was detected in six isolates whereas the transposon Tn*554*, which comprises the genes *met*, *erm*(A), *spc* and three transposase genes, was found in five isolates (Fig. 2). The upstream regions of the complete transposons Tn*558* and Tn*554* in *E. faecalis* are closely related (Fig. 2). Moreover, the genomic regions flanking *optrA* are similar to those of *E. faecalis* strains SS85 and E016 reported in previous studies^29,31^.

**Figure 3:**
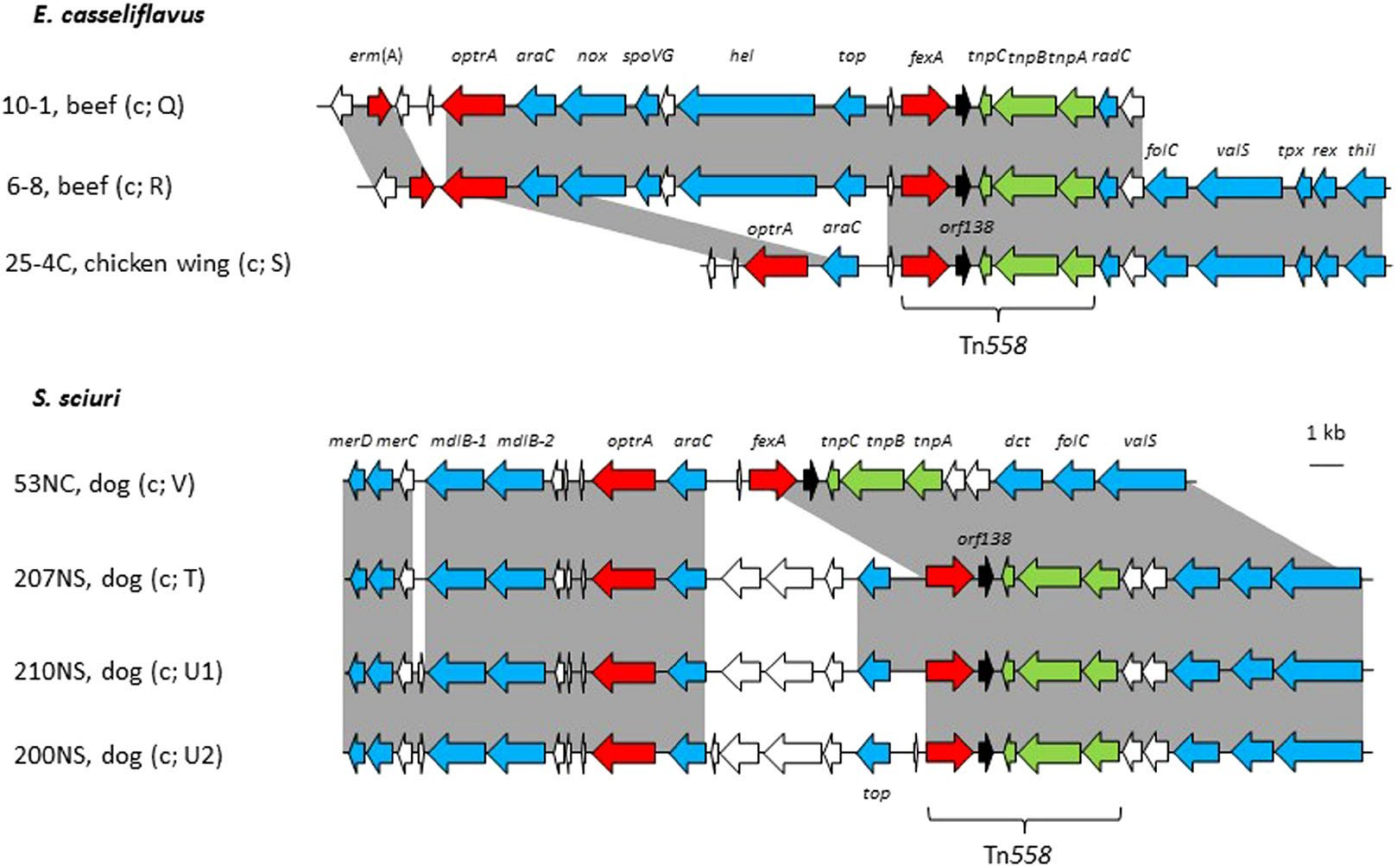
Schematic representation of the genetic environment of chromosomally-located *optrA* in the three *E. casseliflavus* and four *S. sciuri* isolates investigated in this study. The information in the brackets includes the location of the gene *optrA* (c, chromsomal DNA) and the PFGE type, both according to Table 1. Shaded areas represent regions of >99% nucleotide sequence identity. White arrows represent genes coding for hypothetical proteins, red arrows those coding for antimicrobial resistance genes, green arrows those coding for transposases, and blue arrows those coding for other known functions. Genes with known functions coded for a putative NADH oxidase (*nox*), the septation protein SpoVG (*spoVG*), a helicase (*hel*), a topoisomerase (*top*), a C4-dicarboxylate ABC transporter permease (*dct*), a folylpolyglutamate synthase (*folC*), a valine-tRNA ligase (*valS*), a thiol peroxidase (*tpx*), a redox-sensing transcriptional repressor (*rex*), and a thiamine-phosphate kinase (*thil*), proteins involved in mercury resistance (*merC* and *merD*), and a multidrug ABC transporter (*mdlB-1* and *mdlB-2*). The *radC* gene codes for a DNA repair protein and represents the preferential chromosomal integration site for Tn*558*.

The chromosomal *optrA* region of two *E. casseliflavus* isolates (10-1 and 6-8) closely corresponded to that of three *E. faecalis* isolates (11-7, 11-8 and 67AC). In contrast, the segment carrying the genes *nox*, *spoVG*, *hel* and *top*, which is located between *araC*-*optrA* and Tn*558* is missing in the third *E. casseliflavus* isolate (25-4C) (Fig. 3).

The chromosomal *optrA* regions of the four *S. sciuri* isolates 31, 207NS, 210NS, and 200NS were very similar and contained the core *araC-optrA* segment and Tn*558*^32^. The region of isolate 31 differed from the others by the absence of the topoisomerase gene *top* and three reading frames for hypothetical proteins (Fig. 3). The genetic relationships of the *E. casseliflavus* and *S. sciuri* isolates were also investigated by SNP analysis (Supplementary Fig. 4).

### Relatedness of the isolates

**Figure 4:**
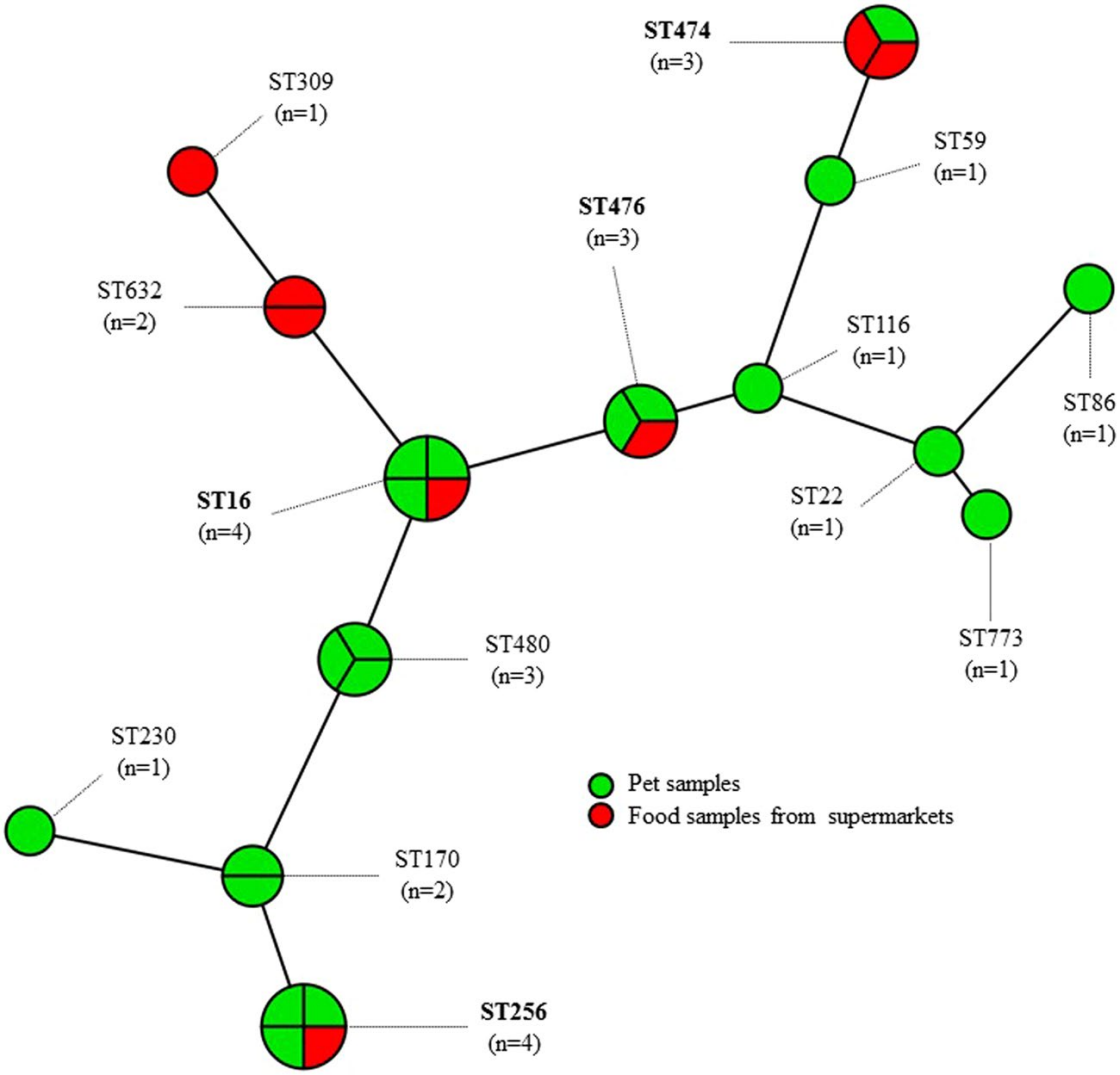
Minimum spanning tree by MLST type of *optrA*-positive *E. faecalis* from companion animals and food samples from supermarkets in Beijing. Each node within the tree represents a single ST. Length of branches between each node represents the number of different alleles (out of seven MLST genes) that differ between two linked nodes/ST. Selected nodes are labelled with corresponding ST and number of isolates represented. MLST = multilocus sequence typing. ST = sequence type.

PFGE analysis of the 28 *E. faecalis* isolates revealed 16 patterns (Table 1, Supplementary Fig. 5), whereas each of the three *E. casseliflavus* isolates exhibited a distinct pattern and the four *S. sciuri* isolates showed three patterns (Table 1, Supplementary Fig. 6), suggesting a high diversity among the *optrA*-positive isolates (Table 1). Moreover, the results of genetic relationships between 28 *E. faecalis* isolates by MLST analysis and SNP analysis were consistent. Nevertheless, closely related PFGE patterns were observed for two *E. faecalis* isolates from nasal samples of dogs (121NS, 233NC) and one *E. faecalis* isolate from beef (3–8), for two *E. faecalis* isolates from anal and nasal samples of dogs (52AC, 203NC) and one isolate (22-4) from a chicken wing, as well as for one isolate (207AE) from an anal sample of a dog and two *E. faecalis* isolates (11-7, 11-8) from egg and beef samples from supermarkets. However, in two of the three paired groups of isolates, differences in either the location of the *optrA* gene and/or the resistance patterns were observed. Nevertheless, one group of paired isolates, *E. faecalis* 52AC and 203NC from two unrelated dogs and *E. faecalis* 22-4 from a chicken wing revealed the chromosomal location of the *optrA* gene, had the same MLST type and *optrA* genetic environment, and showed the same resistance patterns. Moreover, the observation that closely related *E. faecalis* isolates 8-2 and 8-3, 11-7 and 11-8 with the same MLST types (Table 1, Fig. 4) and resistance patterns and an *optrA* plasmid of similar size and similar *optrA* genetic environment (Table 1, Figs 1 and 2) were found among different food items originating from the same supermarket, points towards a possible cross-contamination at this supermarket. Several pairs of canine *E. faecalis* (100AC and 114AC; 190AC and 192NC; 72AC and 37AC) were identified which shared the same MLST types (except for 100AC and 114AC which share 6/7 housekeeping gene alleles), and were closely related in their PFGE patterns, their resistance patterns and the location of the *optrA* gene (Table 1, Figs 1 and 4). Since all samples originated from individual dogs, the occurrence of such related *optrA*-positive isolates may be explained by the contact between the dogs (and/or their excrements). In this regard, contacts between the dogs in the waiting area of the China Agricultural University Veterinary Teaching Hospital need also be taken into account. Even though most closely related *E. faecalis* isolates from dogs and food products were detected in only one case, it suggests that transmission of *optrA*-positive *E. faecalis* between contaminated food products and dogs may occur.

### WGS accession numbers

The WGS of all isolates investigated in this study have been deposited at GenBank under accession numbers: VRVJ00000000 (*E. faecalis* 182NS), VRVK00000000 (*E. faecalis* 114AC), VRVL00000000 (*E. faecalis* 67AC), VRVM00000000 (*E. faecalis* 61NC), VRVN00000000 (*E. faecalis* 131AC), VRVO00000000 (*E. faecalis* 203NC), VRVP00000000 (*E. faecalis* 99AE), VRVQ00000000 (*S. sciuri* 200NS), VRYP00000000 (*E. casseliflavus* 6-8), VWNH00000000 (*S. sciuri* 207NS), VWNI00000000 (*S. sciuri* 210NS), VWOD00000000 (*S. sciuri* 53NC), VWNJ00000000 (*E. faecalis* 75AC), VWNK00000000 (*E. faecalis* 109AC), VWNL00000000 (*E. faecalis* 8-3), VWNM00000000 (*E. faecalis* 11-8), VWNN00000000 (*E. faecalis* 3-8), VWNO00000000 (*E. faecalis* 11-7), VWNP00000000 (*E. faecalis* 22-4), VWNQ00000000 (*E. faecalis* 27-3C), VWNR00000000 (*E. faecalis* 52AC), VWNS00000000 (*E. faecalis* 68AC), VWNT00000000 (*E. faecalis* 72AC), VWNU00000000 (*E. faecalis* 82AC), VWNV00000000 (*E. faecalis* 100AC), VWNW00000000 (*E. faecalis* 121NS), VWNX00000000 (*E. faecalis* 190AC), VWNY00000000 (*E. faecalis* 192NC), VWNZ00000000 (*E. faecalis* 207AE), VWOA00000000 (*E. faecalis* 233NC), VWOE00000000 (*E. faecalis* 37AC), VWOF00000000 (*E. faecalis* 5-6), VWOG00000000 (*E. faecalis* 8-2), VWOB00000000 (*E. casseliflavus* 10-1), VWOC00000000 (*E. casseliflavus* 25-4C).

## Conclusion

This is – to the best of our knowledge – the first large-scale screening study for the presence of *optrA*, *poxtA* and *cfr* in enterococci and staphylococci isolated from companion animals and supermarket-derived vegetable and meat samples. The wide distribution of *optrA* in bacteria of these samples is of great concern for public health, especially when these bacteria are involved human infections. It is noteworthy that some *E. faecalis* isolates from food products and/or companion animals are closely related in their molecular and phenotypic characteristics, highlighting the possibility of bacterial spread between fresh foods from supermarkets and dogs. Because of their frequent and close contact with companion animals, we assume that bacteria – including oxazolidinone-resistant ones – can be exchanged between humans and companion animals in either direction. Further surveillance and control efforts are needed to reduce *optrA*-positive bacteria in both companion animals and food products at supermarkets.

All data generated or analysed during this study are included in this article (and its Supplementary Information Files).

## Methods

### Ethics statement

Sampling procedures were approved by the China Agricultural University Laboratory Animal Welfare and Animal Experimental Ethical Inspection Committee, and were carried out in accordance with the Committee’s approved guidelines.

### Bacterial isolation and detection of resistance genes and mutations

Anal and nasal swab samples were collected from healthy pets (239 dogs and 11 cats) at the Veterinary Teaching Hospital of the China Agricultural University in Beijing. The fact that the dogs and cats included in this study were fed with raw meat, vegetables, or industrial food was assessed by a questionnaire, in which also the type of food items and the supermarket from which it was purchased were requested. Samples of bacteria from the surfaces of vegetables and meat purchased from the aforementioned supermarkets were collected by submerging the foodstuffs in buffered peptone water. Samples of bacteria from industrial food were collected by submerging the foodstuffs in buffered peptone water immediately after the pet food package was opened. Samples were plated on BHI medium and incubated at 37 °C for 24 h. Bacterial species identification was conducted by matrix-assisted laser desorption/ionization time-of-flight (MALDI-TOF) mass spectrometry analysis and 16S rRNA gene sequencing. Phenicol-resistant bacteria were selected on brain heart infusion agar plates containing 8 µg/ml florfenicol. All florfenicol-resistant isolates were screened for the presence of *cfr/cfr*(B)/*cfr*(C), *fexA*, *fexB*, *optrA*, *poxtA*, and mutations in 23S rRNA or in the genes coding for ribosomal proteins L3, L4, and L22 accounting for oxazolidinone resistance by PCR and sequence analysis using previously described primers^12,14,19,33–35^.

### Antimicrobial susceptibility testing

The susceptibility of *optrA*-positive isolates to ten antimicrobial agents was determined using the broth microdilution method^36^. Results were interpreted according to Clinical and Laboratory Standards Institute criteria laid down in documents VET08 (florfenicol, chloramphenicol, ampicillin, erythromycin)^36^ and M100-S28 (vancomycin, linezolid, tedizolid, daptomycin, minocycline, ciprofloxacin)^37^.

### Molecular analyses

The *optrA*-carrying isolates were subjected to SmaI macrorestriction analysis with subsequent PFGE. PFGE results were analyzed using BioNumerics (version 5.1; Applied Maths, Austin, TX, USA). The definition of a PFGE cluster was based on a similarity cutoff of 80%. The location of *optrA* was analyzed by S1-nuclease PFGE and Southern blot analysis^19^. MLST of the *optrA*-positive *E. faecalis* isolates was done by searching the assembled contigs of the recognized chromosomal DNA sequences, and by specific PCR assays if necessary. The primers used for this approach were those indicated at http://efaecalis.mlst.net/: Sequence types (STs) and corresponding MLST gene allele profiles were entered into BioNumerics (Applied Maths, Belgium).

### Genome sequencing analysis

DNA was extracted from all *optrA*-positive isolates using a TIANamp Bacteria DNA Kit (Tiangen Biotech Co., Beijing, China) according to the manufacturer’s instructions. Potential DNA degradation and contamination were analysed by electrophoresis of aliquots in 1% agarose gels. The DNA purity was checked by using the NanoDrop UV-Vis Spectrophotometer (Thermo Fisher Scientific, Shanghai, China). DNA libraries were prepared using the KAPA Hyper Prep Kit (Roche, Basel, Switzerland) and sequenced on an Illumina HiSeq X Ten platform with 150-bp paired-end reads by Berry Genomics Company (Beijing, China). The draft genomes were assembled using SPAdes version 3.9.0 (http://cab.spbu.ru/software/spades/).

The draft genomes were used for the analysis of the genetic environment of *optrA* on plasmids of different sizes or in the chromosomal DNA of the *Enterococcus* and *Staphylococcus* isolates. To determine whether recombination between two IS*1216E* elements can result in the formation of *optrA*-carrying minicircles in isolates that carried the gene *optrA* on a plasmid, inverse PCR assays were conducted using outward primers (primer 1: 5′-CCAGCACCCTGAACCATTCT-3′; primer 2: 5′-CCGGTGTCCTCTTTGTCAGG-3′; annealing temperature 58 °C). A draft assembly of the sequences was generated using CLC Genomics Workbench 9 (CLC Bio, Aarhus, Denmark), the assembly algorithm of which uses de Bruijn graphs^38^. All contigs with an average coverage of >100-fold were screened for the presence of *optrA* using BLAST analysis and LRE-Finder^23^. The regions flanking *optrA*-carrying contigs were identified by primer walking^19^. Sequence analysis was conducted using ORF Finder (http://www.ncbi.nlm.nih.gov/gorf/gorf.html) and BLAST analysis (https://blast.ncbi.nlm.nih.gov/Blast.cgi). Based on draft genomes, core-genome SNP-based phylogenetic trees of *optrA*- positive enterococci and staphylococci belonging to the same species were constructed using Parsnp in the Harvest package^39^ with the default parameter settings and then visualized using iTOL^40^.

## Supplementary information

Supplementary Dataset 1
