# Supplementary material for: Analysis of combined resistance to oxazolidinones and phenicols among bacteria from dogs fed with raw meat/vegetables and the respective food items
Source: Sci Rep. 2019 Oct 29;9:15500. doi: 10.1038/s41598-019-51918-y (PMC6820769; doi:10.1038/s41598-019-51918-y)
Supplement: Supplementary file 1 — Supplementary Dataset 1 [file 41598_2019_51918_MOESM1_ESM.docx]

**Supplemental Material**

**Analysis of combined resistance to oxazolidinones and phenicols among bacteria from dogs fed with raw meat/vegetables and the respective food items**

Yifan Wu^1^, Run Fan^1^, Yinchao Wang^1^, Lei Lei^1^, Andrea T. Feßler^2^, Zheng Wang^1^, Congming Wu^1^, Stefan Schwarz^1,2,**^, Yang Wang^1,*^

^1^ Beijing Advanced Innovation Center for Food Nutrition and Human Health, College of Veterinary Medicine, China Agricultural University, Beijing, 100193, PR China

^2^ Institute of Microbiology and Epizootics, Center for Infection Medicine, Department of Veterinary Medicine, Freie Universität Berlin, 14163 Berlin, Germany

**Supplementary Table 1.** MICs for all tested antibiotics except linezolid and tedizolid.

| Isolate | FFC | CHL | VAN | DPC | MIN | AMP | ERY | CIP | VIR | GEN | RIF | CLI | TIA | OXA |
| --- | --- | --- | --- | --- | --- | --- | --- | --- | --- | --- | --- | --- | --- | --- |
|  |  |  |  |  |  |  |  |  |  |  |  |  |  |  |
| *E. faecalis* |  |  |  |  |  |  |  |  |  |  |  |  |  |  |
| 72AC | 128 | 128 | 4 | 4 | 16 | 1 | >256 | 1 | 32 | ≤500 | 2 | - | - | - |
| 61NC | 128 | 128 | 4 | 2 | 16 | 2 | >256 | 32 | 32 | >1000 | 4 | - | - | - |
| 67AC | 64 | 64 | 2 | 2 | 16 | 1 | >256 | 16 | 32 | >1000 | 1 | - | - | - |
| 82AC | 128 | 128 | 4 | 4 | 32 | 1 | >256 | 1 | 16 | ≤500 | 1 | - | - | - |
| 100AC | 128 | 32 | 4 | 2 | 16 | 2 | 64 | 1 | 16 | ≤500 | 1 | - | - | - |
| 192NC | 64 | 32 | 4 | 2 | 16 | 1 | >256 | 2 | 32 | >1000 | 4 | - | - | - |
| 233NC | 128 | 128 | 4 | 4 | 32 | 1 | >256 | 16 | 32 | >1000 | 1 | - | - | - |
| 182NS | 64 | 32 | 4 | 1 | 16 | 1 | >256 | 2 | 16 | >1000 | 4 | - | - | - |
| 68AC | 64 | 16 | 2 | 1 | 16 | 1 | >256 | 32 | >32 | >1000 | 1 | - | - | - |
| 99AE | 64 | 64 | 4 | 4 | 32 | 1 | >256 | 16 | 32 | >1000 | 1 | - | - | - |
| 37AC | 128 | 128 | 2 | 2 | 16 | 1 | >256 | 1 | 16 | ≤500 | 1 | - | - | - |
| 131AC | 64 | 16 | 4 | 4 | 16 | 2 | >256 | 32 | 32 | >1000 | 2 | - | - | - |
| 114AC | 128 | 32 | 4 | 4 | 16 | 1 | 32 | 1 | 32 | ≤500 | 1 | - | - | - |
| 190AC | 64 | 32 | 2 | 2 | 16 | 1 | 256 | 2 | 16 | >1000 | 4 | - | - | - |
| 52AC | 16 | 32 | 2 | 2 | 32 | 2 | >256 | 32 | 16 | >1000 | 2 | - | - | - |
| 75AC | 128 | 128 | 4 | 2 | 32 | 1 | 32 | 2 | 32 | >1000 | 2 | - | - | - |
| 109AC | 32 | 64 | 2 | 1 | 8 | 1 | >256 | 32 | 32 | >1000 | 2 | - | - | - |
| 121NS | 64 | 64 | 2 | 4 | 32 | 1 | >256 | 16 | 32 | >1000 | 1 | - | - | - |
| 207AE | 64 | 16 | 4 | 4 | 16 | 1 | >256 | 0.5 | >32 | >1000 | 1 | - | - | - |
| 203NC | 16 | 64 | 2 | 2 | 32 | 1 | >256 | 32 | 32 | >1000 | 2 | - | - | - |
| 11-7 | 16 | 64 | 1 | 2 | 16 | 2 | >256 | 16 | 32 | >1000 | 4 | - | - | - |
| 11-8 | 16 | 64 | 1 | 1 | 16 | 2 | >256 | 32 | 32 | >1000 | 4 | - | - | - |
| 3-8 | 32 | 16 | 1 | 2 | 8 | 1 | 8 | 32 | 16 | ≤500 | 1 | - | - | - |
| 22-4 | 64 | 64 | 1 | 1 | 16 | 1 | >256 | 32 | 32 | >1000 | 1 | - | - | - |
| 8-2 | 64 | 64 | 1 | 2 | 16 | 1 | 4 | 16 | 32 | ≤500 | 4 | - | - | - |
| 8-3 | 128 | 128 | 1 | 2 | 16 | 2 | 4 | 32 | 32 | ≤500 | 4 | - | - | - |
| 27-3C | 64 | 32 | 1 | 2 | 16 | 1 | >256 | 2 | 32 | >1000 | 1 | - | - | - |
| 5-6 | 64 | 64 | 1 | 4 | 16 | 2 | 0.25 | 64 | 16 | ≤500 | 0.5 | - | - | - |
|  |  |  |  |  |  |  |  |  |  |  |  |  |  |  |
| *E. casseliflavus* |  |  |  |  |  |  |  |  |  |  |  |  |  |  |
| 10-1 | 32 | 64 | 8 | 1 | 16 | 1 | >256 | 16 | 4 | ≤500 | 4 | - | - | - |
| 6-8 | 16 | 16 | 8 | 8 | 8 | 2 | 0.25 | 1 | 4 | ≤500 | 4 | - | - | - |
| 25-4C | 32 | 64 | 8 | 8 | 8 | 1 | >256 | 1 | 4 | ≤500 | 0.02 | - | - | - |
|  |  |  |  |  |  |  |  |  |  |  |  |  |  |  |
| *S. sciuri* |  |  |  |  |  |  |  |  |  |  |  |  |  |  |
| 207NS | 128 | 32 | 4 | 2 | 8 | 0.25 | 256 | 0.5 | 4 | 0.064 | 0.02 | 0.5 | 16 | 0.5 |
| 200NS | 128 | 64 | 2 | 2 | 0.12 | 0.25 | 0.25 | 1 | 4 | 0.064 | 0.02 | 0.5 | 64 | 0.5 |
| 210NS | 128 | 64 | 4 | 2 | 0.12 | 0.25 | 0.25 | 2 | 4 | 0.064 | 0.02 | 0.5 | 64 | 32 |
| 31 | 128 | 64 | 2 | 2 | 1 | 2 | >256 | 1 | 8 | 4 | 0.02 | 8 | 32 | >32 |

MICs of antibiotics in µg/mL; CHL, chloramphenicol; FFC, florfenicol; LZD, linezolid; TZD, tedizolid; MIN, minocycline; ERY, erythromycin; GEN, gentamicin; DPC, daptomycin; CLI, clindamycin; TIA, tiamulin; AMP, ampicillin; CIP, ciprofloxacin; RIF, rifampicin; VIR, virginiamycin M1; -, absence of tests.

**Supplementary Table 2.** Prevalence (%) of other antibiotic resistance phenotypes among the 31 *optrA*-positive florfenicol-resistant enterococci.

| Origin | Antimicrobial agents^a^ | | | | | | | | | | | |
| --- | --- | --- | --- | --- | --- | --- | --- | --- | --- | --- | --- | --- |
|  | CHL | LZD | TED | MIN^b^ | ERY^c^ | CIP | VIR | GEN | DPC | RIF | VAN | AMP |
| Dogs (n=20) | 100 | 60 | 65 | 100 | 100 | 50 | 10 | 75 | 0 | 20 | 0 | 0 |
|  |  |  |  |  |  |  |  |  |  |  |  |  |
| Food (n=11) | 100 | 36.4 | 72.7 | 72.7 | 54.5 | 72.7 | 0 | 36.4 | 18.2 | 54.5 | 0 | 0 |
|  |  |  |  |  |  |  |  |  |  |  |  |  |
| P value | 1.0000 | 0.2734 | 1.0000 | 0.0367 | 0.0027 | 0.2755 | 0.5269 | 0.0564 | 0.1183 | 0.1055 | 1.0000 | 1.0000 |

^a^ CHL, chloramphenicol; LZD, linezolid; TZD, tedizolid; MIN, minocycline; ERY, erythromycin; GEN, gentamicin; DPC, daptomycin; AMP, ampicillin; CIP, ciprofloxacin; RIF, rifampicin; VIR, virginiamycin M1; VAN, vancomycin.

^b^ Resistance rates differed significantly between samples from pets and supermarkets by Fisher's exact test (*p*<0.05).

^c^ Resistance rates differed significantly between samples from pets and supermarkets by Fisher's exact test (*p*<0.01).

**
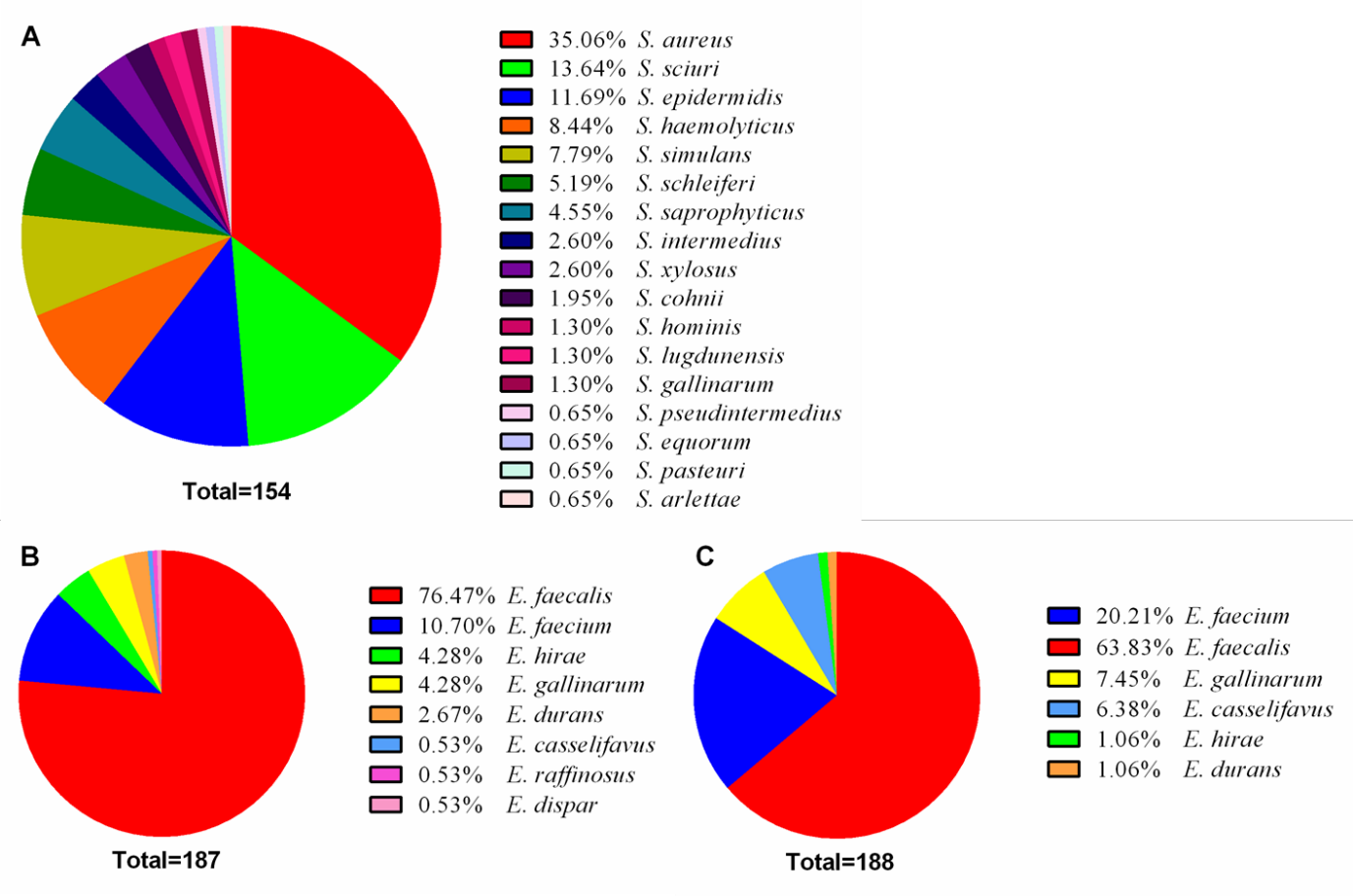
**

**Supplementary Fig. 1.** Prevalence (%) of different subspecies of *Staphylococcus* and *Enterococcus* isolated from pets and food items. (A) Staphylococci isolated from pets, (B) enterococci isolated from pets, and (C) enterococci isolated from food items at supermarkets.


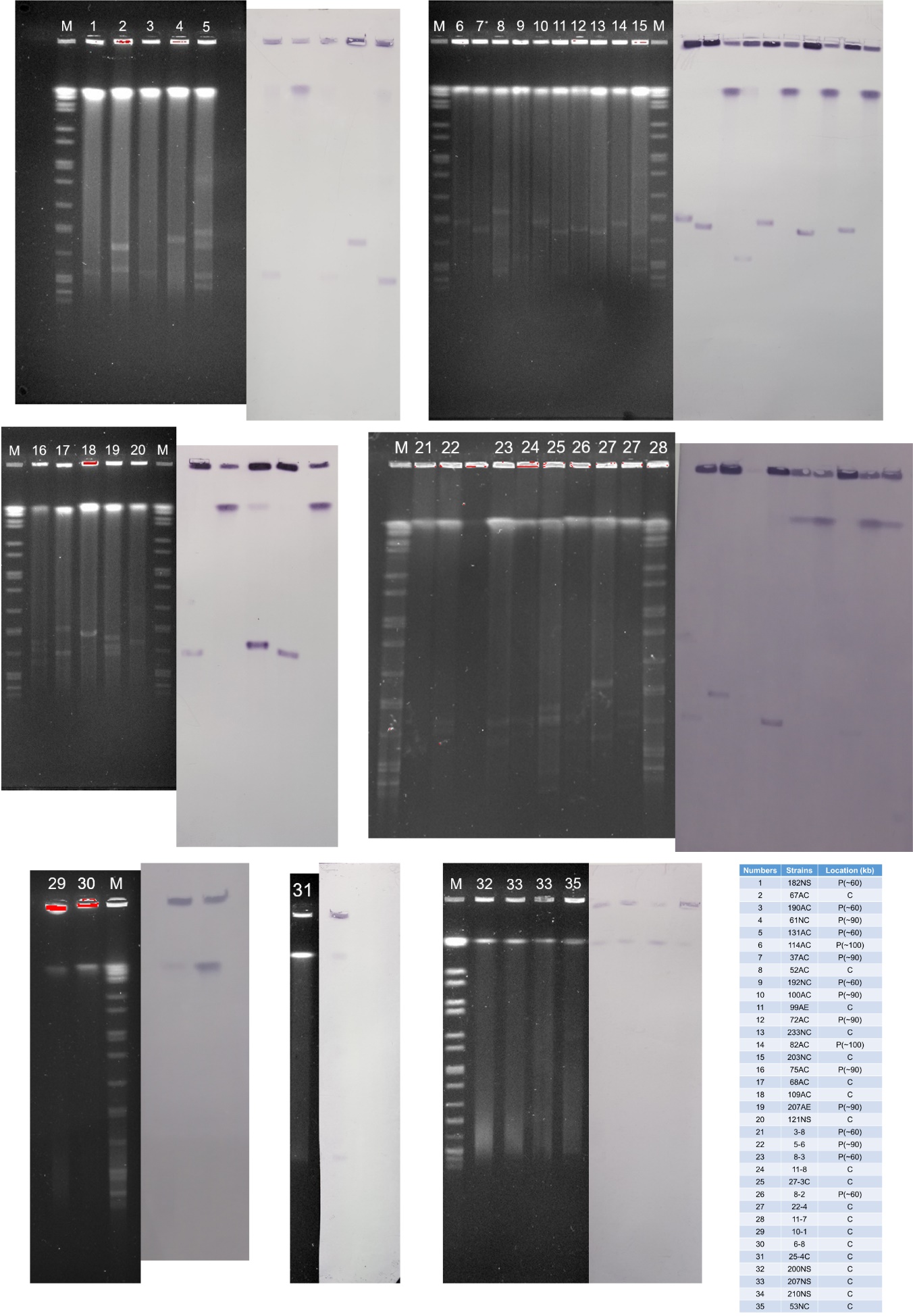


**Supplementary Fig. 2.** S1-nuclease PFGE and Southern blot analysis of *optrA*-positive florfenicol-resistant isolates. Marker, *Salmonella* Braenderup strain H9812.


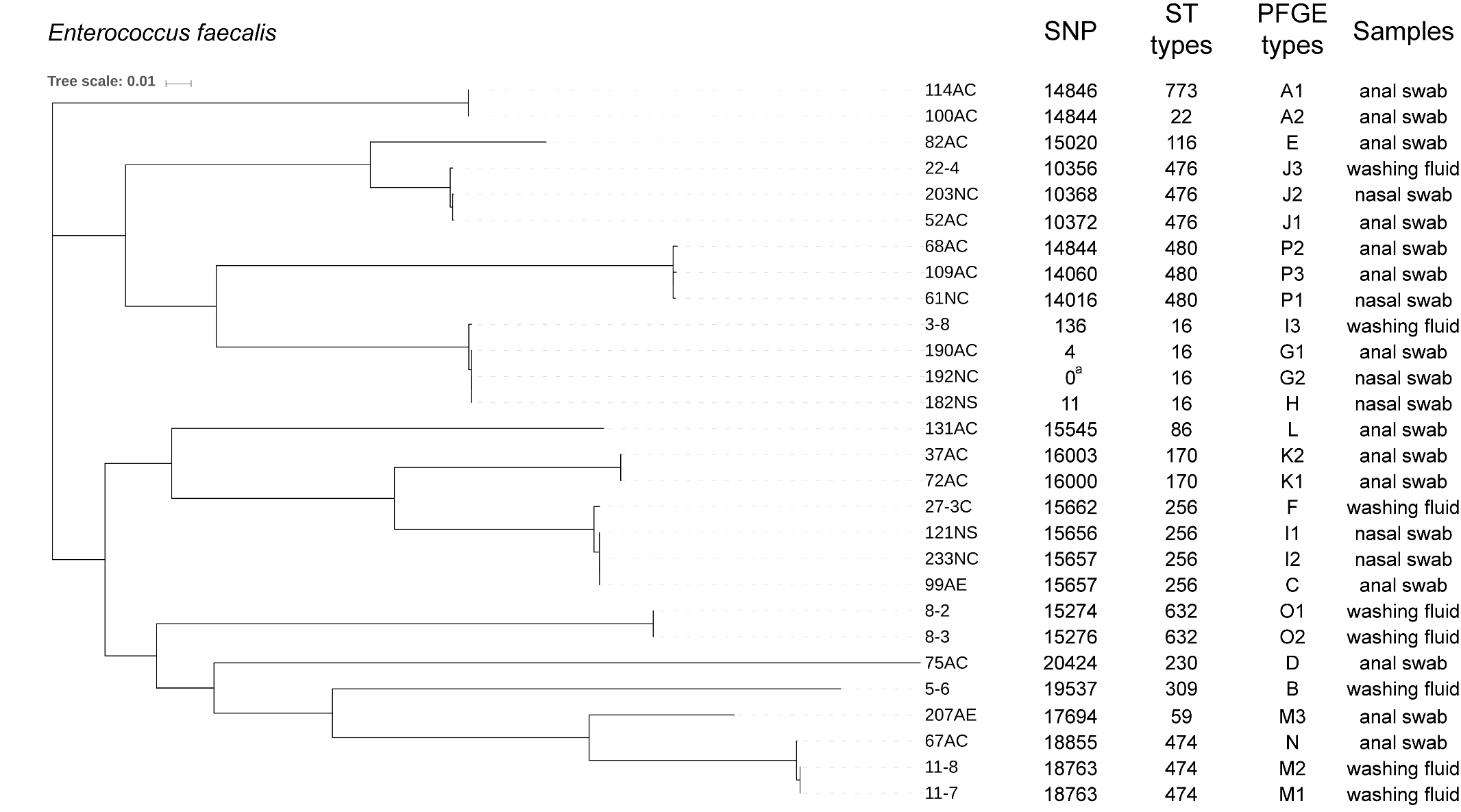


**Supplementary Fig. 3.** Single nucleotide polymorphism analysis tree of 28 *E. faecalis* isolates. ^a^, reference genome.


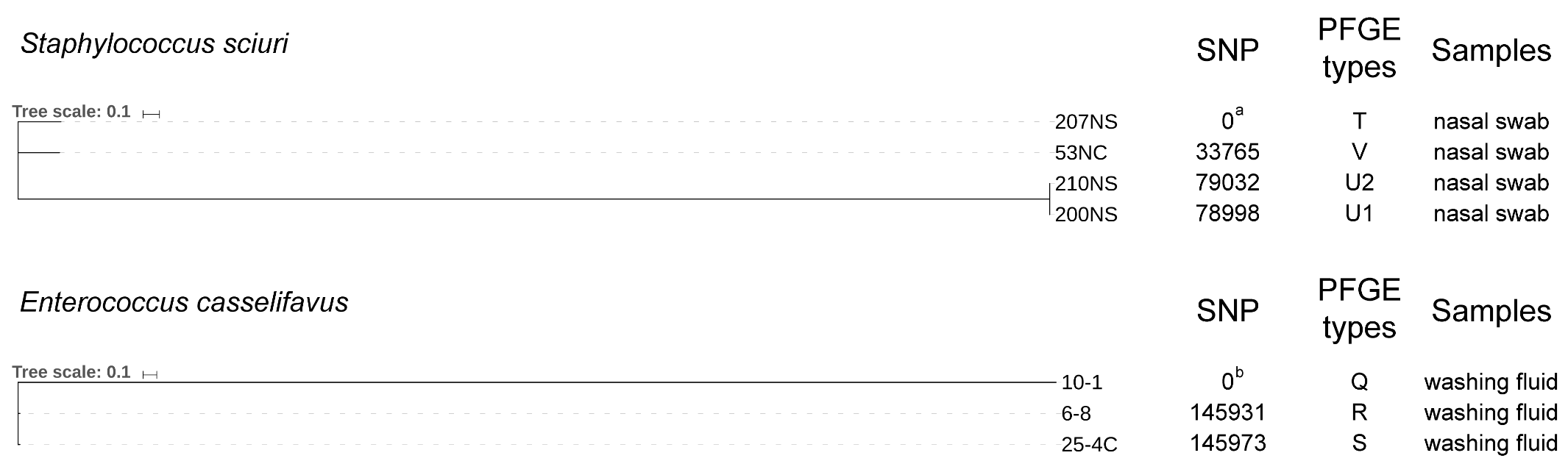


**Supplementary Fig. 4.** Single nucleotide polymorphism analysis tree of 4 *S. sciuri* isolates and 3 *E. casselifavus* isolates. ^a^, ^b^, reference genome.


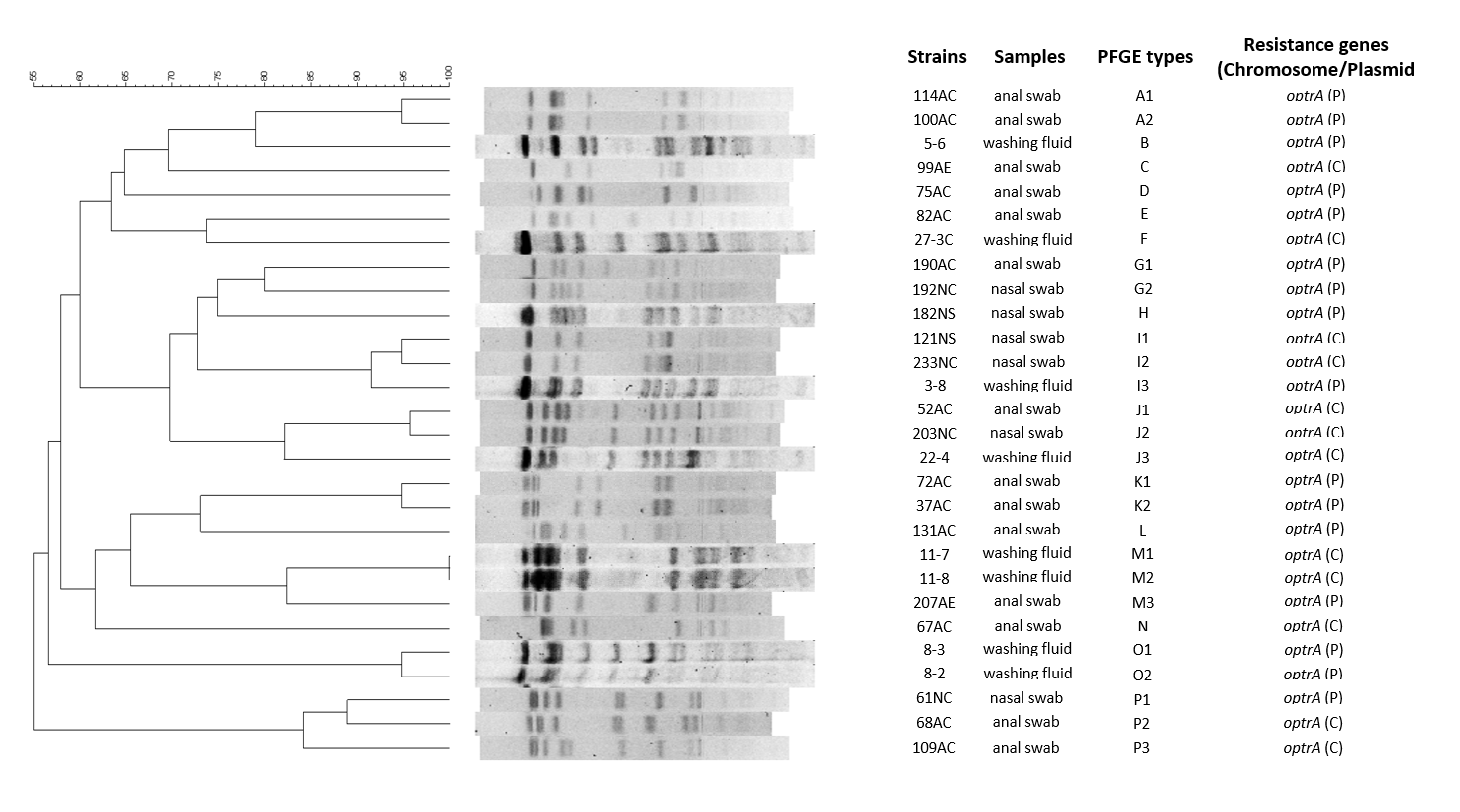


**Supplementary Fig. 5.** Pulsed-field gel electrophoresis (PFGE) patterns of *Sma*I-digested DNA extracted from the 35 *optrA*-positive florfenicol-resistant *E. faecalis* isolates.


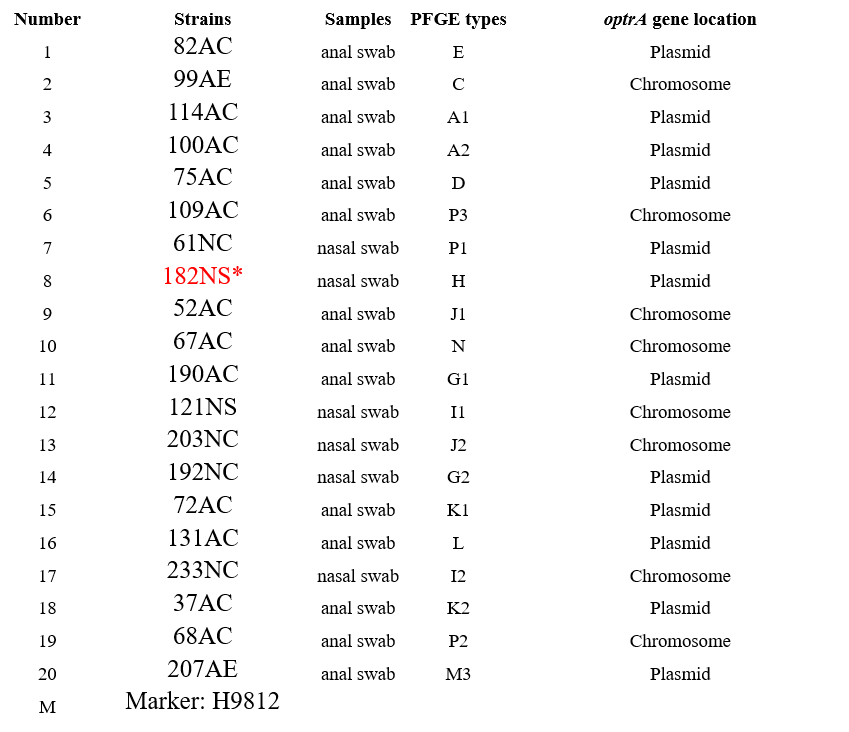
**Original PFGE gels used for supplemental Fig. 5**


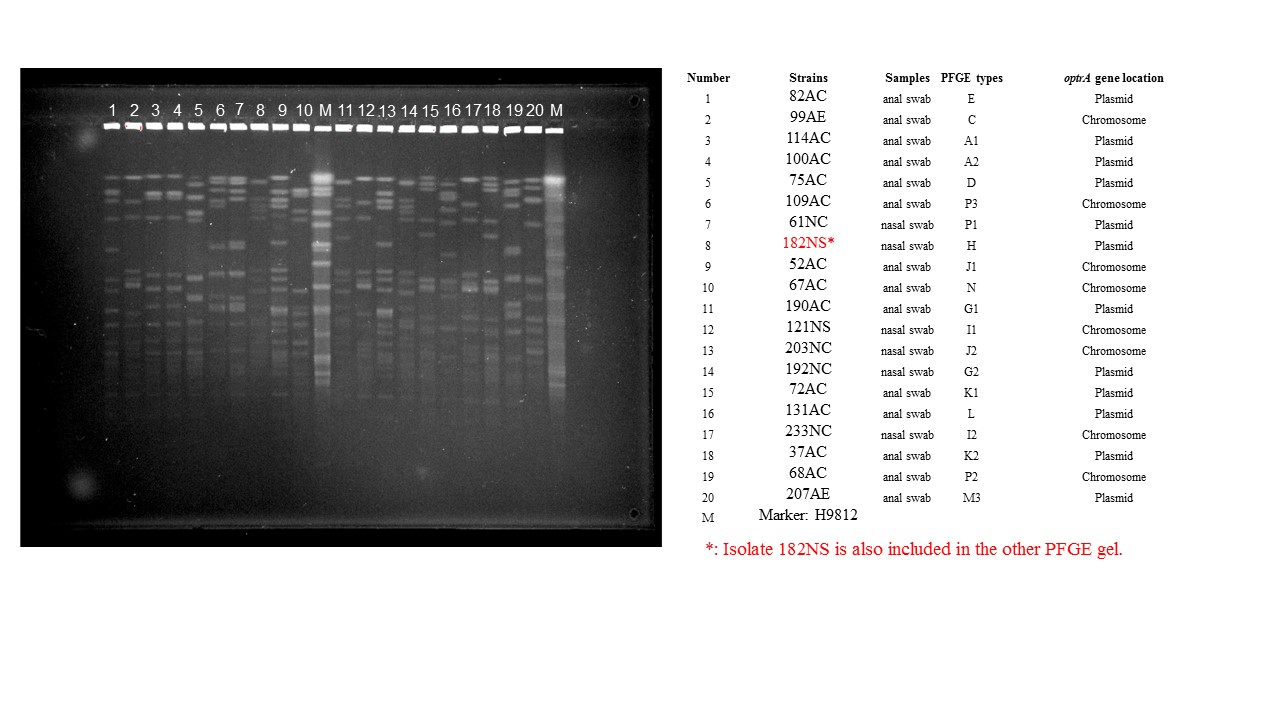


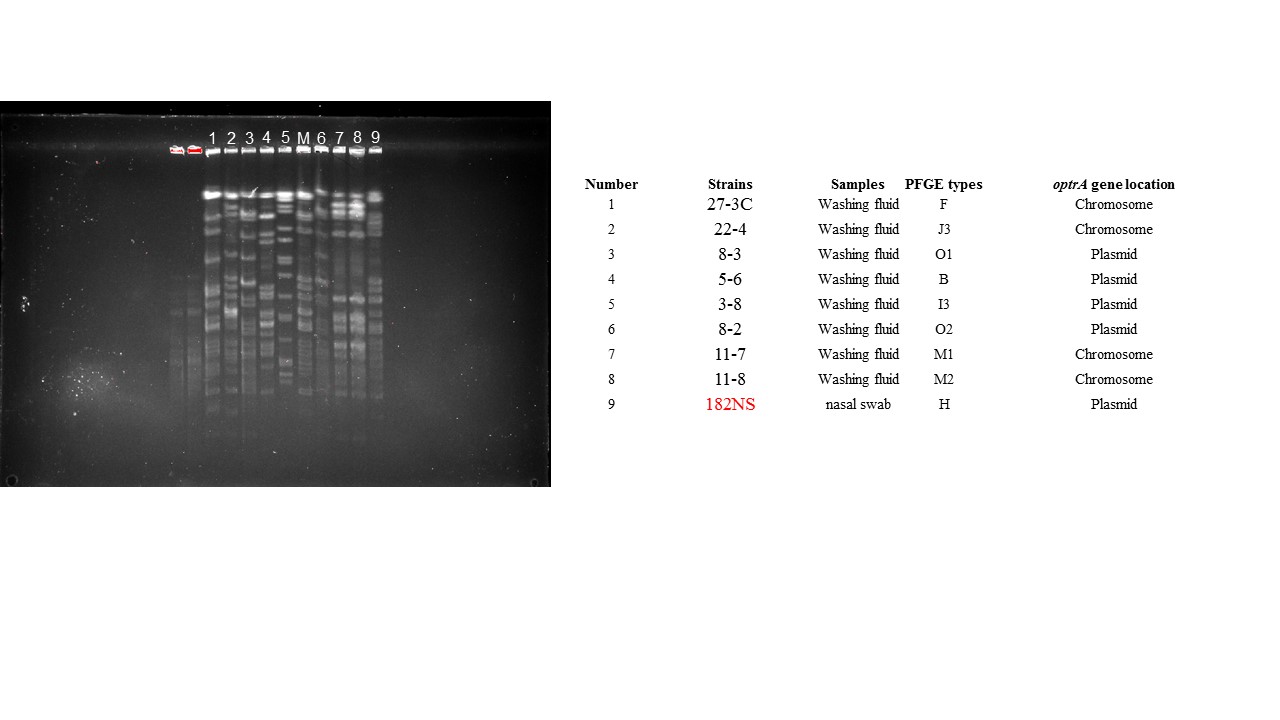


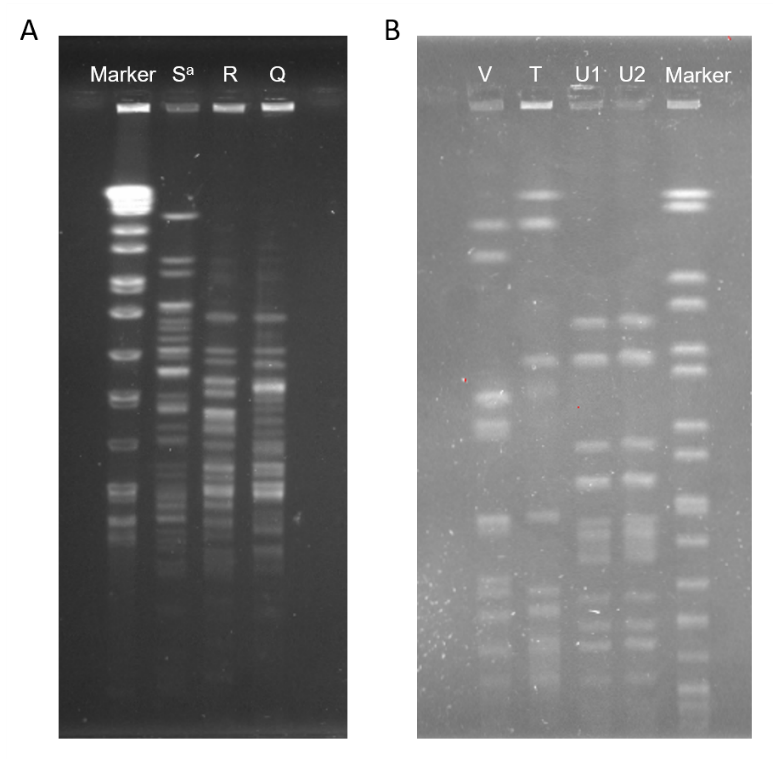


**Supplementary Fig. 6.** Pulsed-field gel electrophoresis (PFGE) patterns of *Sma*I-digested DNA extracted from the seven *optrA*-positive florfenicol-resistant *E. casseliflavus* (A), and *S. sciuri* (B) isolates. Marker, *Salmonella enterica* strain H9812; ^a^, PFGE patterns.
